# Results for Nonlinear Diffusion Equations with Stochastic Resetting

**DOI:** 10.3390/e25121647

**Published:** 2023-12-12

**Authors:** Ervin K. Lenzi, Rafael S. Zola, Michely P. Rosseto, Renio S. Mendes, Haroldo V. Ribeiro, Luciano R. da Silva, Luiz R. Evangelista

**Affiliations:** 1 Departamento de Física, Universidade Estadual de Ponta Grossa, Ponta Grossa 84030-900, PR, Brazil; michelyrosseto@gmail.com; 2 National Institute of Science and Technology for Complex Systems, Centro Brasileiro de Pesquisas Físicas, Rio de Janeiro 22290-180, RJ, Brazil; luciano@fisica.ufrn.br; 3 Departamento de Física, Universidade Tecnológica Federal do Paraná, Apucarana 86812-460, PR, Brazil; rzola1@kent.edu; 4 Departamento de Física, Universidade Estadual de Maringá, Maringa 87020-900, PR, Brazil; rsmendes@dfi.uem.br (R.S.M.); hvr@dfi.uem.br (H.V.R.); lre@dfi.uem.br (L.R.E.); 5 Departamento de Física, Universidade Federal do Rio Grande do Norte, Natal 59078-900, RN, Brazil; 6 Istituto dei Sistemi Complessi (ISC–CNR), Via dei Taurini, 19, 00185 Rome, Italy

**Keywords:** Tsallis entropy, *q*-exponentials, anomalous diffusion, Lévy distributions

## Abstract

In this study, we investigate a nonlinear diffusion process in which particles stochastically reset to their initial positions at a constant rate. The nonlinear diffusion process is modeled using the porous media equation and its extensions, which are nonlinear diffusion equations. We use analytical and numerical calculations to obtain and interpret the probability distribution of the position of the particles and the mean square displacement. These results are further compared and shown to agree with the results of numerical simulations. Our findings show that a system of this kind exhibits non-Gaussian distributions, transient anomalous diffusion (subdiffusion and superdiffusion), and stationary states that simultaneously depend on the nonlinearity and resetting rate.

## 1. Introduction

**Figure 1:**
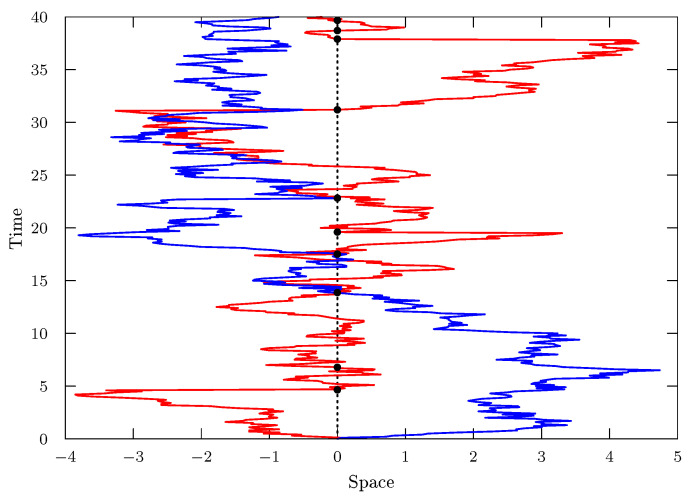
This figure illustrates the stochastic resetting process. The red and blue lines represent the stochastic motion of two particles, which after some time restart the motion (black points) with some rate.

Stochastic processes are one of the most captivating occurrences in the natural world and significantly impact various contexts. Diffusion completely depends on these processes, determining the type of diffusion the system manifests. For example, Markovian processes are typical of the Brownian motion characterized by a linear dependence on the mean square displacement, i.e., $\langle {\left(\Delta x\right)}^{2}\rangle ∼t$, and can be connected with the Gaussian distribution. On the other hand, the non-Markovian processes can be connected to extensions of the Brownian motion where the sub- or super-diffusion is present. In these cases, we have a nonlinear time dependence on the mean-square displacement, e.g., $\langle {\left(\Delta x\right)}^{2}\rangle ∼t^{\sigma }$, where σ<1 and σ>1 correspond to sub- and superdiffusion, respectively. Other behaviors for the mean-square displacement are also possible, such as $\langle {\left(\Delta x\right)}^{2}\rangle ∼\ln^{\sigma }t$, which characterize an ultraslow diffusion. Behind each of these processes, we have a density of probability, which is the solution of the differential equation related to the type of stochastic process present in the system. The usual diffusion is connected to Markovian processes, which have the Gaussian distribution as a solution. Several kinds of differential equations can emerge in the context of non-Markovian processes. The porous media equation is one of them, as a consequence of a Langevin equation with multiplicative noise [1,2] with implications in different contexts [3,4]. It is given by

(1) $$\begin{array}{c} \frac{\partial }{\partial t}\rho \left(x,t\right)=D\frac{\partial^{2}}{\partial x^{2}}\rho^{\nu }\left(x,t\right)\;, \end{array}$$

where *D* is the diffusion coefficient and ρ(x,t) represents the probability distribution of finding a particle around position *x* at time *t*. Equation (1) can be obtained by using different approaches, such as the ones present in Refs. [1,5,6]. This equation has been successfully applied in many situations such as heavy-ion collisions [7], climate modeling, particles with repulsive power-law interactions [8], life sciences [9], and hydrological setting [10]. Further, it can be related to the Tsallis formalism [11] and connected to the thermostatistic aspects [12], similar to the standard diffusion equation and the Boltzmann–Gibbs statistics. These scenarios and others related to stochastic processes are part of the diffusion phenomena, which can be found in different contexts and are essential mechanisms in nature. The diffusion can often appear combined with different phenomena such as stochastic resetting [13,14], a process in which particles are stochastically repositioned to their initial positions at a constant rate (see, for example, Figure 1).

Examples of systems with stochastic reset include the production of proteins by ribosomes [15], visual working memory in humans [16], protein identification in DNA [17], and animal foraging [18]. Motivated by this myriad of possible applications, several works have systematically investigated the combination of diffusion with stochastic resetting [19,20,21,22,23,24,25,26]. Other phenomena that are often combined with diffusion are the reaction–diffusion processes, which play an essential role in different contexts such as physics [4,27,28] and biology [29,30]. Despite this increasing interest in studying diffusion with stochastic resetting and reaction processes, much less attention has been paid to considering nonlinear diffusion processes.

Here, we help to fill this gap by investigating a diffusive process governed by a nonlinear diffusion equation with stochastic resetting and linear reaction processes, both irreversible and reversible. We consider the diffusion governed by Equation (1), a nonlinear equation whose solutions are distributions asymptotically characterized by a compact or a long-tailed behavior. In the last case, we can relate them with the Lévy distributions [31,32], characterized asymptotically by power laws. The results that emerge from this context combine different processes, i.e., the nonlinear diffusion, which may exhibit compact or long-tailed solutions, reaction terms, and stochastic resetting. It is also worth mentioning that the nonlinear diffusion equation considered here can be connected to unusual characteristics such as fractal and multifractal properties, e.g., present in a porous media. The reaction terms can be used to simulate different situations. One of them is the case where the substrate can immobilize the particles. Another can result in an intermittent motion, where the terms are related to the motion and pause while the diffusion proceeds [27]. We perform analytical and numerical analysis for this nonlinear diffusion process with stochastic resetting and reaction terms. In particular, we found an analytical solution for the stationary state when reaction terms are absent, in terms of the *q*-exponential, which has a power-law behavior. The analytical solution for the stationary state is also obtained when a reversible reaction process is considered. These solutions, given in terms of the *q*-exponentials [33], are different from the standard cases discussed in Refs. [19,34]. This feature can be connected to the diffusion process, which is governed by a nonlinear diffusion equation instead of the usual one and results in a correlated anomalous diffusion [35,36].

The remainder of this manuscript is organized as follows. Section 2 defines the diffusion equation, presents the approach to finding its solution, and describes the probability distribution of the positions of particles and the mean square displacement for stochastic resetting for different scenarios. This section also considers the first passage-time distribution and reaction terms for the nonlinear diffusion process with resetting. Finally, Section 3 concludes this work with an overview of our main findings.

## 2. Nonlinear Diffusion Equation with Stochastic Resetting

Let us start our analysis by considering a system subjected to the following diffusion equation:

(2) $$\begin{array}{c} \frac{\partial }{\partial t}\rho \left(x,t\right)=D\frac{\partial^{2}}{\partial x^{2}}{\left[\rho (x,t)\right]}^{\nu }-r\left[\rho \left(x,t\right)-\delta \left(x-x^{\prime }\right)\right]\;,\; \end{array}$$

where ρ(x,t) represents the probability distribution of finding a particle around position *x* at time *t*, *r* is the rate under which particles stochastically reset their positions to $x^{\prime }$, ν is a parameter associated with the properties of the media, and *D* is a constant corresponding to the usual diffusion coefficient. It is worth mentioning that we will also consider some extensions of Equation (2) and implications for the reset process. The solution of this equation in the absence of the resetting term, that is, for r=0, can be found in terms of the *q*-exponential present in the Tsallis formalism [11], which is based on the following entropy:

(3) $$\begin{array}{c} S_{q}=\frac{k}{q-1}\left\{1-\int dx{\left[\rho \left(x,t\right)\right]}^{q}\right\}\;,\; \end{array}$$

where *q* represents a degree of nonextensivity and *k* is a constant. Equation (3) recovers the Boltzmann–Gibbs entropy in the limit of q→1. In particular, it is possible to show that the solution is given by

(4) $$\begin{array}{c} \rho \left(x,t\right)=\frac{1}{\Phi (t)}\exp_{q}\left[-\frac{k^{\prime }x^{2}}{2\left(2-q\right)D\Phi^{2}\left(t\right)}\right]\;,\; \end{array}$$

where q=2−ν and the *q*-exponential is defined as follows:

(5) $$\begin{array}{c} \exp_{q}\left[x\right]=\left\{\begin{array}{cc} {\left[1+(1-q)x\right]}^{\frac{1}{1-q}} & \;,x\geq 1/(q-1)\\ 0 & \;,x<1/(q-1) \end{array}\right.\;,\; \end{array}$$

and $\Phi \left(t\right)={\left[\left(1+\nu \right)k^{\prime }t\right]}^{\frac{1}{1+\nu }}$, with

(6) $$\begin{array}{c} k^{\prime }=2\nu D\pi \left\{\begin{array}{cc} \frac{1}{1-\nu }{\left[\frac{\Gamma \left(\frac{1}{1-\nu }-\frac{1}{2}\right)}{\Gamma \left(\frac{1}{1-\nu }\right)}\right]}^{2} & \;,\nu <1\\ \frac{1}{\nu -1}{\left[\frac{\Gamma \left(\frac{1}{\nu -1}-1\right)}{\Gamma \left(\frac{1}{\nu -1}+\frac{3}{2}\right)}\right]}^{2} & \;,\nu >1 \end{array}\right.\;.\; \end{array}$$

The mean square displacement for this case is given by $\sigma_{x}^{2}\left(t\right)=\langle {\left(x-\langle x\rangle \right)}^{2}\rangle \propto t^{2/(1+\nu )}$, which implies that depending on the values of ν, sub, normal, or superdiffusion can be obtained. Another interesting point about these solutions is their connection with the Lévy distributions for q>1 or ν<1 as discussed in Refs. [31,32].

Equation (2) may be obtained from a random walk approach for r≠0, similar to the standard case [37,38]; however, with a nonlinear dependence to obtain the nonlinearity present in the diffusive term. To proceed this way, we follow the approach of Ref. [39], yielding

(7) $$\begin{array}{ccc} \rho (x,t+\tau ) & = & \int_{-\infty }^{\infty }e^{-r\tau }\Psi \left[x-x^{\prime },t;\rho \left(x-x^{\prime },t\right)\right]\rho \left(x-x^{\prime },t\right)\Phi \left(x^{\prime }\right)dx^{\prime }\\ & + & \left\{1-\Psi [x,t;\rho (x,t)]\right\}e^{-r\tau }\rho \left(x,t\right)+\left.\left(1-e^{-r\tau }\right)\{\rho \left(x,t\right)-R\left[\rho (x,t)\right]\right\}\;.\; \end{array}$$

By taking the limits τ→0 and $x^{\prime }\to 0$ as discussed in Ref. [39], it is possible to simplify Equation (7) and obtain

(8) $$\frac{\partial }{\partial t}\rho \left(x,t\right)=\left.\frac{\partial^{2}}{\partial x^{2}}\{\Psi \left[x,t;\rho \left(x,t\right)\right]\rho \left(x,t\right)\right\}-rR\left[\rho (x,t)\right]\;,\;$$

which for $\Psi \left[x,t;\rho \left(x,t\right)\right]=D{\left[\rho \left(x,t\right)\right]}^{\nu -1}$ and $R\left[\rho (x,t)\right]=\left(\rho \left(x,t\right)-\delta \left(x-x^{\prime }\right)\right)$ recovers Equation (2). In fact, replacing the previous expressions for Ψ[x,t;ρ(x,t)] and $R\left[\rho (x,t)\right]$ in Equation (8), we obtain the following:

(9) $$\begin{array}{c} \frac{\partial }{\partial t}\rho \left(x,t\right)=D\frac{\partial^{2}}{\partial x^{2}}{\left[\rho (x,t)\right]}^{\nu }-r\left[\rho \left(x,t\right)-\delta \left(x-x^{\prime }\right)\right]\;. \end{array}$$

We notice that Ψ[x,t;ρ(x,t)] directly influences the behavior exhibited by the particles during the diffusion process, which can lead us to normal or anomalous diffusion. It is also possible to consider situations with different regimes of diffusion depending on the expressions used for Ψ[x,t;ρ(x,t)]. Later, we examine a case characterized by two different regimes, i.e., $\Psi \left[x,t;\rho \left(x,t\right)\right]=D_{1}+D_{\nu }{\left[\rho \left(x,t\right)\right]}^{\nu -1}$, where one of the processes is normal and another is anomalous. Formulating the stochastic resetting in terms of a Langevin equation is also possible using the procedure employed in Ref. [21]. To do this, we need to consider the following equation $\dot{x}=\sqrt{\Psi [x,t;\rho (x,t)]}\xi \left(t\right)$, where ξ(t) is a stochastic variable, i.e., a Gaussian white noise [39] with 〈ξ(t)〉=0 and $\langle \xi \left(t\right)\xi \left(t^{\prime }\right)\rangle \propto \delta \left(t-t^{\prime }\right)$. In this manner, Equation (2) (or Equation (8)) can be obtained from a random walk approach with a nonlinear dependence on the probability density function connected to the dynamics of the walkers or employing a stochastic equation with a nonlinear term that is coupled with a nonlinear diffusion equation.

**Figure 2:**
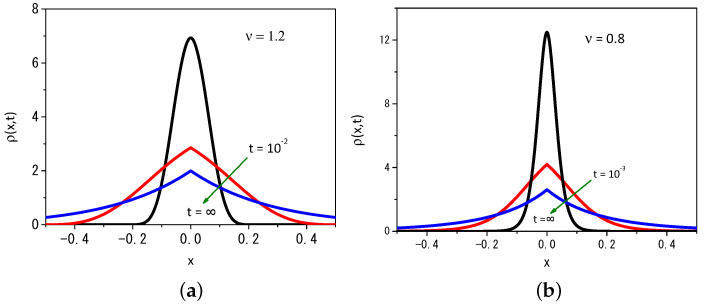
Profile of the distribution obtained from Equation (2) for (**a**) ν=1.2 and (**b**) ν=0.8 by considering different values of time. For illustrative purposes, we consider D=1, ρ(x,0)=δ(x), $x^{\prime }=0$, and r=20.

By performing some numerical calculations, it is possible to find the solution for Equation (2) as shown in Figure 2a,b for ν>1 and ν<1 at three different moments in time. To do this, the system was defined in the interval [−5000, 5000] and discretized in increments of $dx=2\times 10^{-2}$, with $dt=10^{-6}$, to numerically explore the evolution of time and obtain the results exhibited in these figures. These values for dx and dt verify the condition $Ddt/\left(dx^{2}\right)<1/2$ required for the stability of the solutions during the time evolution of the initial condition to satisfy the boundary conditions [28,40].

**Figure 3:**
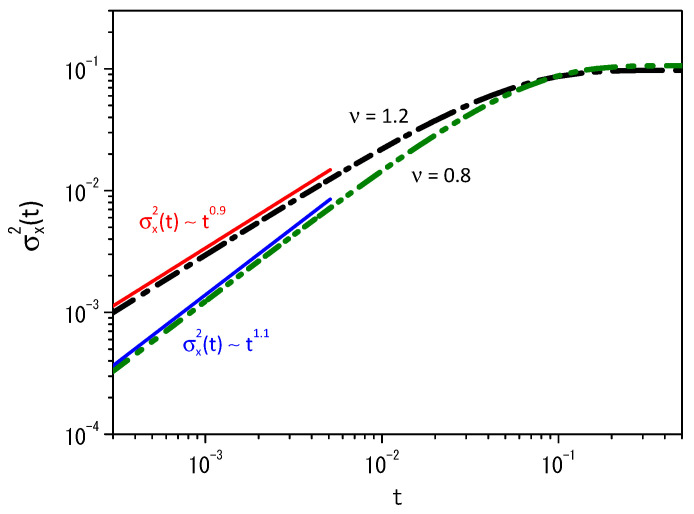
Time-dependence of the mean square displacement, i.e., $\sigma_{x}^{2}\left(t\right)=\langle {\left(x-\langle x\rangle \right)}^{2}\rangle$, obtained from Equation (2) when ν=0.8 (green line) and ν=1.2 (black line). The red and blue lines were incorporated to evidence the behavior of the mean square displacement for short times. Again, for illustrative purposes, we consider D=1, ρ(x,0)=δ(x), $x^{\prime }=0$, and r=20.

Figure 3 exhibits the time-dependence of the mean square displacement for the cases shown in Figure 2a,b. The system reaches a stationary state for long times as in the standard case, i.e., for ν=1, when the resetting is considered. We have an anomalous diffusion for short times in both cases, as shown in Figure 3. We have a superdiffusion for ν<1, whereas the subdiffusion behavior is verified for ν>1.

The result shown in Figure 3 for the stationary state allows us to consider, in the asymptotic limit of t→∞, the following equation:

(10) $$D\frac{\partial^{2}}{\partial x^{2}}{\left[\rho_{st}\left(x\right)\right]}^{\nu }-r\left[\rho_{st}\left(x\right)-\delta \left(x-x^{\prime }\right)\right]=0\;,$$

where $\rho_{st}\left(x\right)=\lim_{t\to \infty }\rho \left(x,t\right)$. It is possible to verify that the solution of Equation (10) is given by

(11) $$\begin{array}{c} \rho_{st}\left(x\right)=\frac{1}{Z}\exp_{q}[-\beta |x-x^{\prime }|], \end{array}$$

with Zβ=2/(2−q), ν=3−2q, and

(12) $$\begin{array}{c} \beta ={\left[\frac{r}{2D\nu }{\left(\frac{2}{2-q}\right)}^{3-2q}\right]}^{\frac{1}{4-2q}}\;. \end{array}$$

**Figure 4:**
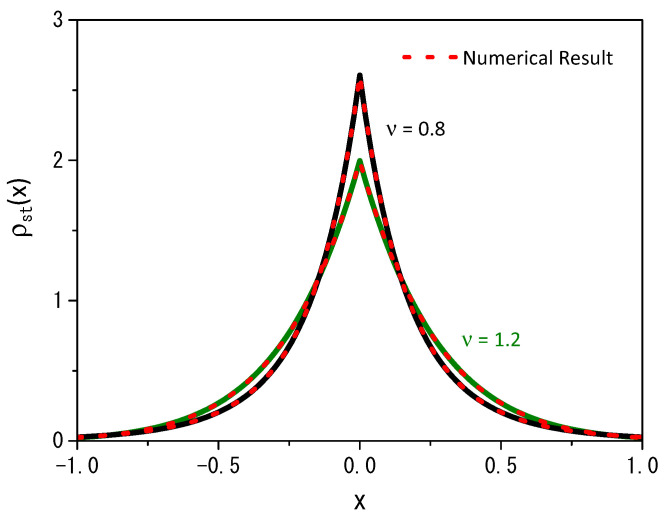
Comparison of the trends of the analytical results (black and green solid lines), given by Equation (11), with the numerical results (red dotted lines), obtained for Equation (2) when ν=0.8 and ν=1.2. As before, the calculations consider D=1, ρ(x,0)=δ(x), $x^{\prime }=0$, and r=20.

Equation (11), for the particular case q=1 (or ν=1), leads to the result obtained in Ref. [14] for the normal case. Figure 4 illustrates the numerical result obtained from Equation (2) for long times, i.e., in the stationary scenario, and the previous analytical result, obtained for Equation (11). It reveals a strong agreement between the numerical and analytical results when we examine two different values of the ν parameter: the analytical result depicted for ν=0.8, with a solid black line and ν=1.2, with a solid green line, while the dotted red line represents the numerical result. In both cases, we employ a stochastic resetting rate of r=20.

We may also consider the survival probability and the first passage time distribution for the situation we are analyzing. To proceed further, we consider the following boundary condition: ρ(0,t)=ρ(∞,t)=0, which implies assuming the presence of an absorbent surface at x=0, and fix, as an initial condition, $\rho \left(x,0\right)=\delta \left(x-x_{0}\right)$. In this framework, Equation (8) becomes

(13) $$\begin{array}{c} \frac{\partial }{\partial t}\rho \left(x,t\right)=D\frac{\partial^{2}}{\partial x^{2}}\rho^{\nu }\left(x,t\right)-r\left[\rho \left(x,t\right)-S\left(t\right)\delta \left(x-x^{\prime }\right)\right]\;, \end{array}$$

where $S\left(t\right)=\int_{0}^{\infty }dx\rho \left(x,t\right)$ is the survival probability. The first passage time distribution can be found by using Equation [41]

(14) $$\begin{array}{c} F\left(t\right)=-\frac{\partial }{\partial t}\int_{0}^{\infty }dx\rho \left(x,t\right)=-\frac{\partial }{\partial t}S\left(t\right)\;. \end{array}$$

**Figure 5:**
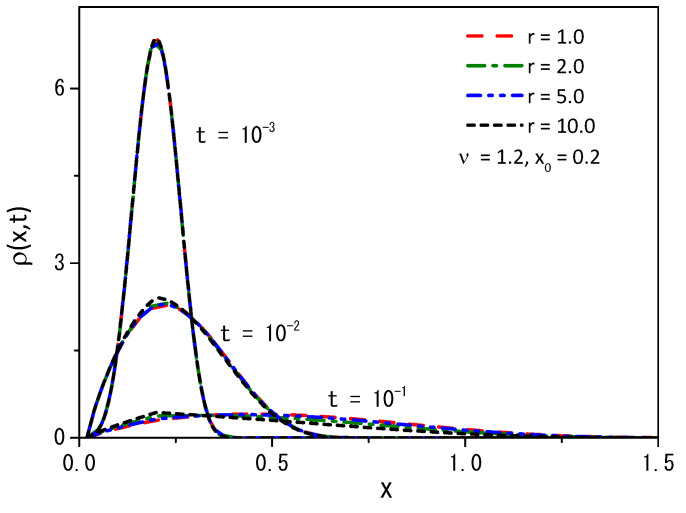
The probability distribution function obtained from Equation (13) when ν=1.2 for the boundary conditions ρ(0,t)=0 and ρ(∞,t)=0. We consider, for simplicity, D=1, $\rho \left(x,0\right)=\delta \left(x-x_{0}\right)$, and $x^{\prime }=x_{0}$.

**Figure 6:**
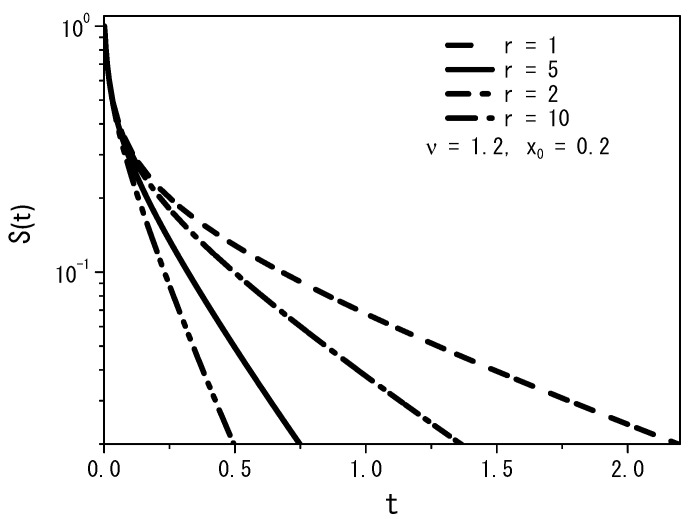
Time-dependence of the survival probability using Equation (13) for various values of the resetting rate *r*, with ν=1.2 for the boundary conditions ρ(0,t)=0 and ρ(∞,t)=0. We consider, again, D=1, $\rho \left(x,0\right)=\delta \left(x-x_{0}\right)$, and $x^{\prime }=x_{0}$.

**Figure 7:**
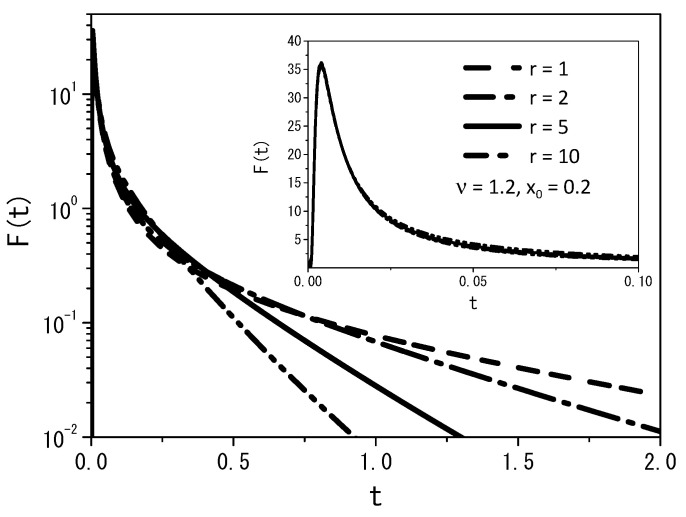
The first passage time distribution obtained from Equation (14) for ν=1.2 and the boundary conditions ρ(0,t)=0 and ρ(∞,t)=0. Again, for simplicity, we consider D=1, $\rho \left(x,0\right)=\delta \left(x-x_{0}\right)$, and $x^{\prime }=x_{0}$.

Figure 5, Figure 6 and Figure 7, for the boundary conditions ρ(0,t)=0 and ρ(∞,t)=0, depict some cases with fixed values of the diffusion coefficient D=1 and position $x^{\prime }=x_{0}$, for ν=1.2 and $\rho \left(x,0\right)=\delta \left(x-x_{0}\right)$.

From Figure 5, we may conclude that the quantity of particles decreases with increasing rate *r*, demonstrating that the particles can find the absorbent surface more easily for large values of the stochastic resetting rate. A similar behavior is illustrated in Figure 6, where we observe the changing dynamics of particle survival probability over time. An increase in the rate parameter *r* corresponds to faster adsorption of the particles at the surface. Figure 7 presents a graph illustrating the first passage time distribution over time, with the curves representing the analytical results obtained from Equation (14).

Another challenging scenario, which emerges when the system is subjected to the resetting process, is represented by the presence of a subtract that immobilizes the particles while the diffusion proceeds. To face this case, we can consider the following equation:

(15) $$\begin{array}{ccc} \rho (x,t+\tau )\;\;\; & = & \;\;\;\int_{-\infty }^{\infty }e^{-\alpha \tau }\Psi \left[x-x^{\prime },t;\rho \left(x-x^{\prime },t\right)\right]\rho \left(x-x^{\prime },t\right)\Phi \left(x^{\prime }\right)dx^{\prime }\\ & + & \;\;\;\left\{1-\Psi [x,t;\rho (x,t)]\right\}e^{-\alpha \tau }\rho \left(x,t\right)+\left.\left(1-e^{-\alpha \tau }\right)\{\rho \left(x,t\right)-R\left[\rho (x,t)\right]\right\}\;. \end{array}$$

From this equation, it is possible to obtain the following diffusion equation:

(16) $$\begin{array}{c} \frac{\partial }{\partial t}\rho \left(x,t\right)=\frac{\partial^{2}}{\partial x^{2}}\{\Psi [x,t;\rho (x,t)]\rho (x,t)\}-r\left[\rho \left(x,t\right)-e^{-\alpha t}\delta \left(x-x^{\prime }\right)\right]-\alpha \rho \left(x,t\right)\;,\; \end{array}$$

in which $\Psi \left[x,t;\rho \left(x,t\right)\right]=D{\left[\rho \left(x,t\right)\right]}^{\nu -1}$ and $R\left[\rho (x,t)\right]=\rho \left(x,t\right)+\left(r/\alpha \right)[\rho \left(x,t\right)$$-e^{-\alpha t}\delta \left(x-x^{\prime }\right)]$. This equation differs from Equation (2) by the presence of a reaction term that immobilizes particles with the rate α. Note that the resetting term considers the exponential $e^{-\alpha t}$ multiplied by the delta function. This factor corresponds to the time behavior of the survival probability in this case. In the absence of the resetting term, it is possible to find the solution in terms of the *q*-exponential as in the previous case, and it is given by

(17) $$\begin{array}{c} \rho \left(x,t\right)=e^{-\alpha t}\frac{1}{\Phi_{\alpha }\left(t\right)}\exp_{q}\left[-\frac{k^{\prime }x^{2}}{2\left(2-q\right)D\Phi_{\alpha }^{2}\left(t\right)}\right]\;, \end{array}$$

with

(18) $$\begin{array}{c} \Phi_{\alpha }\left(t\right)={\left\{\frac{1+\nu }{(1-\nu )\alpha }\left[e^{(1-\nu )\alpha t}-1\right]\right\}}^{\frac{1}{1+\nu }}\;.\; \end{array}$$

In the case of an intermittent motion, we have to consider the following time behavior for $S_{1}\left(t\right)=\left(\alpha_{2}/\alpha_{t}\right)\left(1-\alpha_{2}e^{-\alpha_{t}t}/\alpha_{t}\right)$, where $\alpha_{t}=\alpha_{1}+\alpha_{2}$. In this scenario, the process of resetting and motion is governed by the constants $\alpha_{1}$ and $\alpha_{2}$, and the equations are given by

(19) $$\begin{array}{c} \frac{\partial }{\partial t}\rho_{1}\left(x,t\right)=D\frac{\partial^{2}}{\partial x^{2}}\rho_{1}^{\nu }\left(x,t\right)-r\left[\rho_{1}\left(x,t\right)-S_{1}\left(t\right)\delta \left(x-x^{\prime }\right)\right]-\alpha_{1}\rho_{1}\left(x,t\right)+\alpha_{2}\rho_{2}\left(x,t\right) \end{array}$$

and

(20) $$\begin{array}{c} \frac{\partial }{\partial t}\rho_{2}\left(x,t\right)=\alpha_{1}\rho_{1}\left(x,t\right)-\alpha_{2}\rho_{2}\left(x,t\right)\;.\; \end{array}$$

From an analytical point of view, it is possible to find the solution of the linear case, i.e., ν=1. It is

(21) $$\begin{array}{ccc} \rho_{1}\left(x,t\right) & = & \rho_{0}\left(x,t\right)+\sum_{n=1}^{\infty }{\left(-\alpha_{1}\right)}^{n}\int_{-\infty }^{\infty }dx_{n}\int_{0}^{t}dt_{n}G_{2}\left(x-x_{n},t-t_{n}\right)\\ & \times & \int_{-\infty }^{\infty }dx_{n-1}\int_{0}^{t_{n}}dt_{n-1}G_{2}\left(x_{n}-x_{n-1},t_{n}-t_{n-1}\right)\cdots \\ & \times & \int_{-\infty }^{\infty }dx_{1}\int_{0}^{t_{2}}dt_{1}G_{2}\left(x_{2}-x_{1},t_{2}-t_{1}\right)\rho_{0}\left(x_{1},t_{1}\right) \end{array}$$

and

(22) $$\begin{array}{c} \rho_{2}\left(x,t\right)=\alpha_{1}\int_{0}^{t}dt^{\prime }e^{-\alpha_{2}\left(t-t^{\prime }\right)}\rho_{1}\left(x,t^{\prime }\right)\;,\; \end{array}$$

where

(23) $$\begin{array}{c} \rho_{0}\left(x,t\right)=G_{1}\left(x,t\right)+r\int_{0}^{t}dt^{\prime }S_{1}\left(t^{\prime }\right)G_{1}\left(x,t-t^{\prime }\right)\;,\; \end{array}$$

(24) $$\begin{array}{c} G_{2}\left(x,t\right)=G_{1}\left(x,t\right)+\int_{0}^{t}dt^{\prime }e^{\alpha_{2}t^{\prime }}G_{1}\left(x,t-t^{\prime }\right)\;,\; \end{array}$$

and

(25) $$\begin{array}{c} G_{1}\left(x,t\right)=e^{-rt-x^{2}/\left(4Dt\right)}/\sqrt{4\pi Dt}\;.\; \end{array}$$

**Figure 8:**
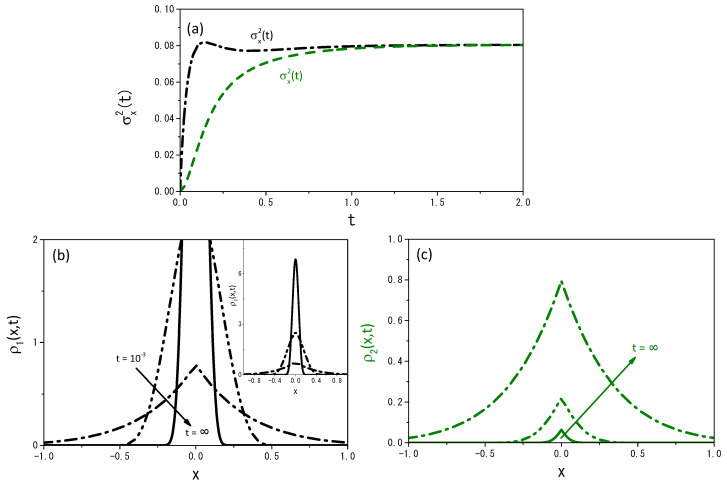
Profiles of the probability distributions obtained from Equations (19) and (20), when ν=1.2. (**a**) exhibits the time-dependence of the mean square displacement for the distributions $\rho_{1}\left(x,t\right)$ and $\rho_{2}\left(x,t\right)$; (**b**,**c**) show the spatial profiles of $\rho_{1}\left(x,t\right)$ and $\rho_{2}\left(x,t\right)$. The curves were drawn for D=1, r=20, $\rho_{1}\left(x,0\right)=\delta \left(x\right)$, $\rho_{2}\left(x,0\right)=0$, and $\alpha_{1}=\alpha_{2}=5$, for illustrative purposes.

**Figure 9:**
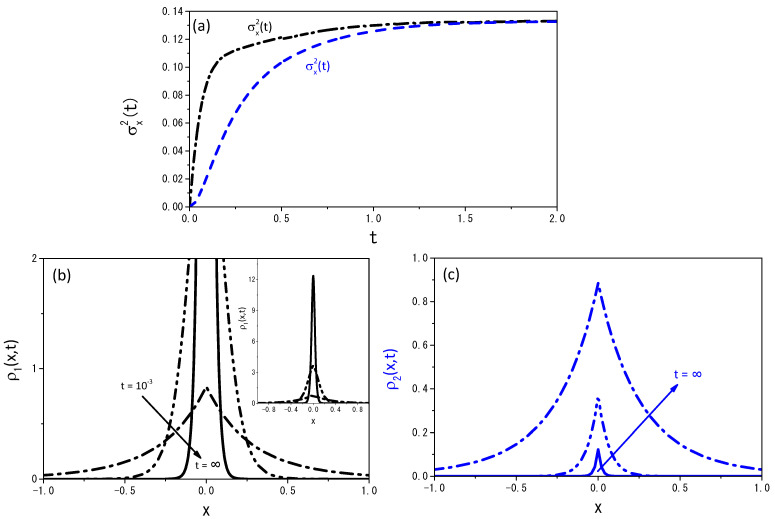
The same as in Figure 8 for ν=0.8.

Figure 8 and Figure 9 illustrate the behavior of the mean square displacement and the distributions obtained from Equations (19) and (20) for different values of ν. For Equations (19) and (20), it is also possible to find the stationary solution, i.e., the one in the limit t→∞. We consider that in this limit, $\alpha_{1}\rho_{1}\left(x,t\right)$ is nearly equivalent to $\alpha_{2}\rho_{2}\left(x,t\right)$, and thus we solve the equation

(26) $$D\frac{\partial^{2}}{\partial x^{2}}{\left[\rho_{1(2),st}\left(x\right)\right]}^{\nu }-r\left[\rho_{1(2),st}\left(x\right)-S_{1(2),st}\delta \left(x-x^{\prime }\right)\right]=0\;,$$

where $\rho_{1(2),st}\left(x\right)=\lim_{t\to \infty }\rho_{1(2)}\left(x,t\right)$ and $S_{1(2),st}=\lim_{t\to \infty }S_{1(2)}\left(t\right)$. It is possible to verify that the solution of Equation (26) is given by

(27) $$\begin{array}{c} \rho_{1(2),st}\left(x\right)=\frac{1}{Z_{1(2)}}\exp_{q}[-\beta_{1}\left|x-x^{\prime }\right|], \end{array}$$

with $Z_{1(2)}\beta_{1(2)}S_{1(2),st}=2/\left(2-q\right)$, ν=3−2q, and

(28) $$\begin{array}{c} \beta_{1(2)}={\left[\frac{r}{2D\nu S_{1(2),st}^{2-2q}}{\left(\frac{2}{2-q}\right)}^{3-2q}\right]}^{\frac{1}{4-2q}}\;. \end{array}$$

**Figure 10:**
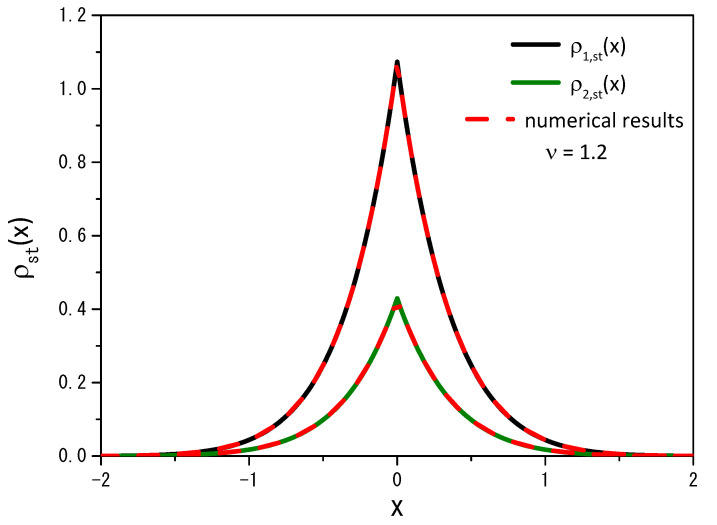
Comparison of the analytical results (black and green solid lines), given by Equation (27), $\rho_{2,st}\left(x\right)=\left(\alpha_{1}/\alpha_{2}\right)\rho_{1,st}\left(x\right)$, with the numerical results (red dotted lines) obtained from Equation (2) for ν=1.2. The curves were drawn for D=1, r=10, $\rho_{1}\left(x,0\right)=\delta \left(x\right)$, $\rho_{2}\left(x,0\right)=0$, $\alpha_{1}=2$, and $\alpha_{2}=5$.

**Figure 11:**
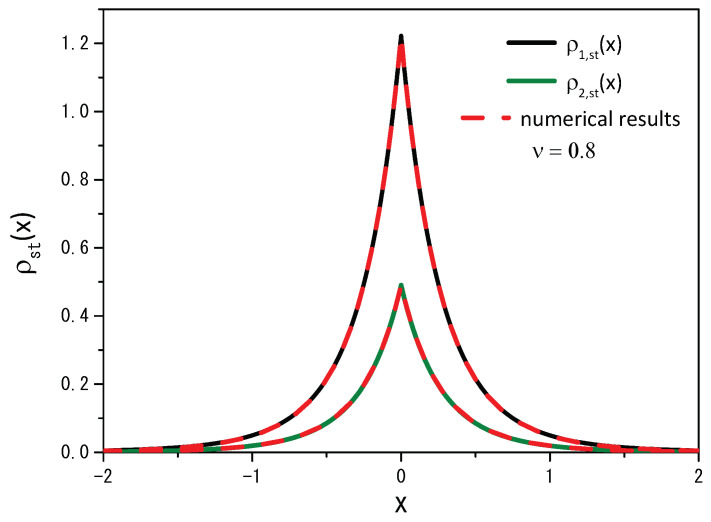
The same as in Figure 10 for the case ν=0.8.

Figure 10 and Figure 11 exhibit the stationary solution for $\rho_{1}\left(x,t\right)$ and $\rho_{2}\left(x,t\right)$ from the numerical and the analytical point of view.

**Figure 12:**
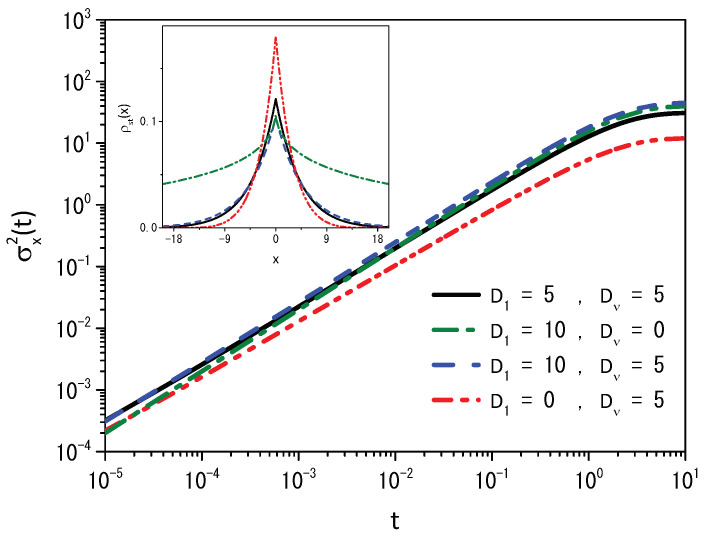
Time-dependence of the mean square displacement obtained from Equation (30) for different values of diffusion coefficients. The inset corresponds to the stationary distribution for different diffusion coefficients. We consider, for illustrative purposes, ν=1.2, $x^{\prime }=0$, and the initial condition ρ(x,0)=δ(x), for r=0.5.

Let us reconsider the random walk approach for r≠0, i.e., Equation (7), given by

(29) $$\begin{array}{ccc} \rho (x,t+\tau ) & = & \int_{-\infty }^{\infty }e^{-r\tau }\Psi \left[x-x^{\prime },t;\rho \left(x-x^{\prime },t\right)\right]\rho \left(x-x^{\prime },t\right)\Phi \left(x^{\prime }\right)dx^{\prime }\\ & + & \left\{1-\Psi [x,t;\rho (x,t)]\right\}e^{-r\tau }\rho \left(x,t\right)+\left.\left(1-e^{-r\tau }\right)\{\rho \left(x,t\right)-R\left[\rho (x,t)\right]\right\}\;, \end{array}$$

which for $\Psi \left[x,t;\rho \left(x,t\right)\right]=D_{1}+D_{\nu }{\left[\rho \left(x,t\right)\right]}^{\nu -1}$ implies that the diffusion is governed by the following equation

(30) $$\frac{\partial }{\partial t}\rho \left(x,t\right)=D_{1}\frac{\partial^{2}}{\partial x^{2}}\rho \left(x,t\right)+D_{\nu }\frac{\partial^{2}}{\partial x^{2}}{\left[\rho \left(x,t\right)\right]}^{\nu }-rR\left[\rho (x,t)\right]\;,$$

with $R\left[\rho (x,t)\right]=\rho \left(x,t\right)-\delta \left(x-x^{\prime }\right)$ in connection with the stochastic resetting. Equation (30) has two different diffusive terms, which allows us to obtain two different regimes, where one of the processes is normal and the other is anomalous. Figure 12 illustrates the behavior of the mean square displacement and stationary distributions obtained from Equation (30) for different values of the diffusion coefficients for ν=1.2. The first regime, which is shown in Figure 12 for the mean square displacement, is anomalous, and the second is normal before reaching the stationary state.

## 3. Discussion and Conclusions

We have investigated the diffusion process governed by a nonlinear diffusion equation when stochastic resetting and linear reaction terms are present. The nonlinear diffusion equation analyzed is the porous media equation with the diffusive part characterized by a single nonlinear term or a combination of different terms, resulting in different diffusion regimes. One of the solutions of Equation (2), in the absence of stochastic resetting and reaction terms, is given in terms of the *q*-Gaussian, as discussed in Section 2. It is different from the normal one expressed in terms of the Gaussian distribution as a consequence of the stochastic processes related to the motion of the particles [1]. It presents an anomalous behavior evidenced by the time dependence of the mean square displacement, which can be connected with sub- and superdiffusion. Under the influence of stochastic resetting, these processes exhibit a stationary state that differs from the expected exponential, characterized by a power-law behavior, as illustrated in Figure 4. This feature is a consequence of the nature of the diffusion process promoted by the nonlinear term, which can be connected to the correlated anomalous diffusion [35,36]. These general results extend the ones obtained in Refs. [19,34]. Subsequently, we analyzed the reaction process in this context by considering an irreversible and reversible scenario. The first case can be related to a substrate that immobilizes the particles while diffusion proceeds. The stationary solution is absent in a different way from the other scenarios. The second case can be considered an intermittent process between the resting and the motion with some rates. For this case, we also obtained a stationary solution in terms of the *q*-exponential, evidencing the influence of the nature of the diffusion on the stochastic resetting. The diffusion process represented by Equation (1) is described by power-law distributions, which promote a different behavior from the normal one for the stochastic resetting and, consequently, a stationary solution expressed in terms of a power-law.

We also analyzed a situation characterized by different diffusion regimes, such that the first regime is slower than the normal one, while the second is faster before the stationary state is reached. We verified that these changes in the diffusion equation directly influence the resetting process, leading the system to exhibit anomalous behavior. These features also open the possibility of considering mixing between different cases, such as the fractional diffusion equations [42,43,44] and nonlinear diffusion equations, which results in fractional nonlinear diffusion equations [45]. Combining different equations will produce a wide class of behaviors to describe a variety of scenarios. Finally, we hope that the results found here can be useful in discussing the processes related to the nonlinear diffusion equation when the stochastic process is present.

## Data Availability

Data are contained within the article.
